# Clinical conceptualisation of PTSD in psilocybin treatment: disrupting a pre-determined and over-determined maladaptive interpretive framework

**DOI:** 10.1177/20451253251342319

**Published:** 2025-06-08

**Authors:** Nadav Liam Modlin, Victoria Williamson, Carolina Maggio, Joanne Stubley, Namik Kirlic, Anthony Cleare, James Rucker

**Affiliations:** The Institute of Psychiatry, Psychology and Neuroscience, King’s College London, 16 De Crespigny Park, London SE5 8AF, UK; South London and Maudsley NHS Foundation Trust, Maudsley Hospital, Denmark Hill, London SE5 8AS, UK; King’s Centre for Military Health Research, King’s College London, London, UK; Department of Psychology, University of Bath, Claverton Down, Bath, UK; The Institute of Psychiatry, Psychology and Neuroscience, King’s College London, London, UK; South London and Maudsley NHS Foundation Trust, Maudsley Hospital, Denmark Hill, London, UK; The Tavistock Trauma Service, The Tavistock and Portman NHS Foundation Trust, London, UK; Compass Pathways, Fora, London, UK; The Institute of Psychiatry, Psychology and Neuroscience, King’s College London, London, UK; South London and Maudsley NHS Foundation Trust, Maudsley Hospital, Denmark Hill, London, UK; The Institute of Psychiatry, Psychology and Neuroscience, King’s College London, London, UK; South London and Maudsley NHS Foundation Trust, Maudsley Hospital, Denmark Hill, London, UK

**Keywords:** psilocybin, psilocybin treatment, psychedelic therapy, psychedelics, PTSD, trauma

## Abstract

Post-traumatic stress disorder (PTSD) and associated trauma and stressor-related disorders are common and debilitating, presenting significant treatment challenges due to their complex interplay of biological, cognitive, affective, somatic and social factors. Current treatments, while advancing and effective, yield limited efficacy for many individuals, underscoring the need for novel therapeutic approaches. This review explores the multifaceted nature of PTSD, emphasising its intricate predisposing and maintaining factors and explores the potential of psilocybin, a classical psychedelic, as a therapeutic agent. This review synthesises recent literature on the safety, efficacy and proposed mechanisms of action and change of psychedelic therapies for psychiatric conditions associated with traumatic stress, including treatment-resistant depression, end-of-life anxiety and anorexia nervosa. Correspondingly, it proposes a conceptual framework for psilocybin treatment in PTSD, framing the condition as a complex, maladaptive interpretive framework that is both predetermined and over-determined. A clinical narrative illustrates how psilocybin’s unique psychopharmacological properties and catalysed subjective effects may facilitate therapeutic progress by disrupting this rigid and restricting framework. Finally, we offer recommendations for the safe administration of psilocybin for traumatised patients in medical research settings, emphasising the importance of rigorous and trauma-informed protocols and comprehensive patient care.

## Introduction

Post-traumatic stress disorder (PTSD) remains a significant global mental health issue, affecting millions.^1^ Initially classified as an anxiety disorder characterised by symptoms of re-experiencing, avoidance, and hyperarousal, PTSD was reclassified under ‘Trauma- and Stressor-Related Disorders’ in the DSM-IV.^2^ This recategorisation underscores the central role of trauma exposure (TE) as an organising factor in the development and maintenance of PTSD, distinguishing it from anxiety disorders and emphasising its unique treatment challenges. The interaction of biological, cognitive, affective, somatic and social processes associated with TE adds complexity to these challenges. Similarly, Complex PTSD (C-PTSD), recognised in the ICD-11, develops from prolonged, repetitive TE, often interpersonal (e.g. forms of childhood abuse, domestic violence.^3^ In addition to PTSD’s core symptoms and dissociative phenotypes, C-PTSD presents with affective dysregulation, negative self-concept and identity, and significant relational dysfunction.^4^ Trauma survivors frequently experience cumulative trauma across their lifespan, leading to widespread biopsychosocial dysfunction that necessitates a nuanced understanding and approach to treatment.^5^

Despite advancements in understanding the development and maintenance of PTSD, effective treatment remains elusive for many patients.^6^ Current first-line, evidence-based psychological treatments include prolonged exposure (PE), trauma-focused cognitive behavioural therapy (CBT) and cognitive processing therapy (CPT), and eye movement desensitisation and reprocessing (EMDR). A recent meta-analysis suggests that 38% of patients accessing psychological treatment for PTSD showed at least a 50% reduction in symptoms.^7^ Specifically, CBT and CPT demonstrate remission rates of approximately 40%–60%. However, treatment dropout rates can be high, with some studies reporting up to 33% of patients not completing treatment.^8^ PE therapy has demonstrated efficacy with remission rates of 30%–50%.^7^ Despite its effectiveness, PE’s intense focus on TE can also lead to dropout rates as high as 40%^8^ whilst EMDR demonstrates remission rates between 30% and 50% with dropout rates of up to 39%.^9^

FDA-approved pharmacotherapies are limited to selective serotonin reuptake inhibitors (SSRIs).^10^ However, these treatments have significant limitations, demonstrating limited efficacy in reducing core symptoms such as re-experiencing, avoidance, and hyperarousal, with remission rates around 20%–30%.^11^ SSRIs have been criticised as being ill-suited for PTSD treatment in general.^12^ Additionally, side effects such as sexual dysfunction, weight gain, and gastrointestinal issues can limit their tolerability and adherence.^13^ SSRIs may also exacerbate symptoms, including insomnia, suicidality and agitation in some patients.^14^ Taken together, many patients experience partial or no response to existing treatments, accompanied by significant treatment dropout rates, underscoring the need for novel treatments.^15^ In response, novel treatment approaches that address not only the core symptoms of PTSD but also the broader spectrum of psychological and social disturbances that arise from TE are required, highlighting the importance of integrating neurobiological, psychological, and social perspectives.^16,17^ Correspondingly, MDMA has emerged as a leading compound in chronic PTSD treatment, earning FDA breakthrough therapy designation and demonstrating robust efficacy in phase-III clinical trials.^18^ When delivered in conjunction with manualised, trauma-focused psychotherapy, it appears to significantly improve treatment outcomes by rapidly reducing fear responses and enhancing emotional openness and trust.^19^ However, approval for MDMA was denied due to concerns over unblinding bias, limited long-term safety data, unclear contributions of psychotherapy, and insufficient risk mitigation for abuse and safety.^20^ Nonetheless, reapplication remains possible with additional supporting evidence.

To date, only one psilocybin treatment in PTSD study has been conducted.^21^ An open-label trial investigating psilocybin for PTSD due to TE in adulthood, top-line data suggests favourable safety and tolerability profile, with initial signs of efficacy. Furthermore, psychedelic forms of therapy have demonstrated safety and efficacy across psychiatric conditions comorbid with traumatic-stress psychopathology,^22^ including end-of-life anxiety,^23^ treatment-resistant depression^24^ and anorexia nervosa.^25^ To explore the potential utility of psilocybin treatment in PTSD, this review explores the multifaceted nature of PTSD, including its predisposing and maintaining factors, the clinical limitations of current treatments, and the potential emerging role of psilocybin and other classical psychedelics. By integrating insights from recent literature, we present a conceptual framework for psilocybin treatment that considers PTSD as a pre-determined and over-determined maladaptive interpretive framework. Correspondingly, we provide a clinical narrative highlighting how psilocybin could contribute to PTSD treatment and provide best practice guidelines for safe administration in research settings.

## Factors associated with the predisposing, developmental and maintaining of PTSD

PTSD arises from a complex interplay of genetic, environmental, psychological and neurobiological factors. Heritability studies suggest a genetic predisposition to PTSD, with polymorphisms in genes such as the serotonin transporter gene contributing to vulnerability.^26^ These genetic factors, along with trauma-induced epigenetic modifications, may dysregulate the hypothalamic-pituitary-adrenal (HPA) axis, impairing stress regulation and increasing susceptibility to PTSD.^27^ Childhood maltreatment, including neglect and abuse, plays a crucial role in shaping this vulnerability. These early adverse experiences may disrupt neurodevelopmental pathways, fostering emotional dysregulation and maladaptive stress responses that heighten the likelihood of developing PTSD later in life.^28^ Adverse childhood experiences (ACEs) act as key psychosocial precipitants, increasing the risk of PTSD by exposing individuals to high levels of stress that interact with pre-existing genetic and neurobiological susceptibilities.^29^

Once PTSD emerges, maladaptive coping strategies such as avoidance, dissociation, and hypervigilance reinforce maladaptive cognitive, behavioural and affective patterns, perpetuating the condition. Initially protective, these responses ultimately hinder emotional processing and adaptation.^30^

Furthermore, a growing body of evidence suggests that intrinsic brain networks, including the default mode network, exhibit disorder-specific alterations that may contribute to the long-term negative effects associated with TE.^31^ These changes contribute to the persistence of hyperarousal, intrusive memories, and avoidance behaviours. More specifically, central to PTSD are dysfunctions within the fear-learning network, comprising the prefrontal cortex (PFC), hippocampus, and amygdala.^32^ Hyperactivity of the amygdala may amplify fear responses and promote overgeneralisation of threats, while hypoactivity in the ventromedial PFC undermines its regulatory control over the amygdala, potentially exacerbating fear responses and emotional dysregulation.^33^ Concurrently, hippocampal dysfunction, including reduced volume and activity, impairs contextual memory processing, potentially adversely influencing patients’ capacity to distinguish between safe and threatening environments.^34^ These disruptions are further compounded by alterations in white matter tracts, such as the cingulum bundle and uncinate fasciculus.^35^ This in turn may diminish connectivity between key brain regions, potentially weakening top-down affective regulation and amplifying fear responses.^35^ Trauma-related biochemical disruptions, including elevated glutamate and reduced N-acetyl-aspartate (NAA) levels in the hippocampus and PFC, may suggest excitotoxic neuronal damage and reduced neural health,^36^ further exacerbating neurocognitive deficits.

Psychosocially, pretrauma variables including gender, age, educational attainment, and baseline cognitive adaptability appear to modulate PTSD risk and burden^37^ with women exhibiting higher prevalence and individuals with reduced mental flexibility demonstrating greater susceptibility. Additionally, peritrauma factors, including trauma nature and severity, degree of dissociation, and perceived threat, influence acute stress responses,^38^ while post-trauma resilience factors, such robust social support, optimism, and adaptive coping skills, mitigate symptom severity and facilitate recovery.^39^

Furthermore, cognitive distortions, such as self-blame, negative beliefs about oneself and the world, and exaggerated perceptions of danger, play a pivotal role in maintaining PTSD, creating an adverse, self-reinforcing cycle that hinders recovery.^40^ These maladaptive cognitive appraisal patterns, combined with behaviours like rumination, thought suppression, and experiential avoidance, intensify distress and further prolong the impact of the condition. Understanding these interconnected factors underscores the importance of targeting neurobiological, cognitive, and psychosocial dimensions in treatment strategies. Furthermore, spiritual beliefs may also intersect with the development and maintenance of PTSD, acting as both a protective and risk factor depending on individual experiences and coping mechanisms.^41^ Positive spiritual frameworks often provide trauma survivors with a sense of coherence, hope, purpose and access to a community, potentially facilitating the integration of fragmented traumatic memories into a meaningful narrative.^42^ This integrative process may reduce distress and foster resilience by counteracting feelings of isolation, helplessness and despair. Conversely, spiritual struggles such as questioning one’s faith following TE can exacerbate self-blame, hopelessness, and existential distress, potentially intensifying PTSD symptoms.^43^

### PTSD as a pre-determined and over-determined maladaptive interpretive framework

As psilocybin and related compounds are being investigated in PTSD and for various indications often comorbid with TE and traumatic stress-related psychopathology^6,22^ the following section provides a conceptual framework to understand the potential clinical utility of psilocybin.

TE may lead to and maintain a sequence of maladaptive and persistent intrapersonal and psychosocial processes. These include dysregulated biological fear responses,^44^ excessive use of psychological defences,^45,46^ development of negative cognitive appraisals and harmful self-narratives^47^ and dysfunctional interpersonal dynamics.^48^ These dimensions interact in a cyclical manner, giving rise to, reinforcing and perpetuating PTSD symptoms. Dysregulated fear responses, for example, involve heightened affective reactivity and threat perception, which in turn may drive excessive reliance on psychological defences such as avoidance, dissociation, or emotional numbing. Correspondingly, these defences may foster negative cognitive appraisals (e.g. self-blame, shame and perceived helplessness) and harmful self-narratives (e.g. traumatic experiences as the central organising feature of one’s self-concept), which can lead to social withdrawal and isolation.

**Figure 1.**
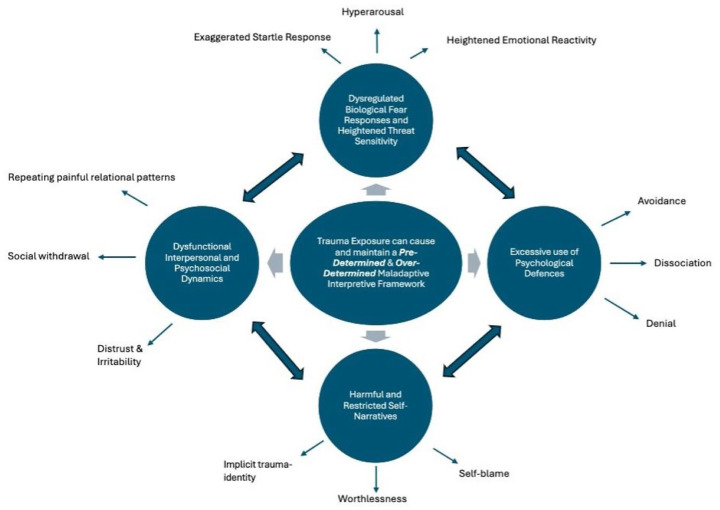
Trauma exposure and the maintenance of maladaptive interpretive frameworks. This figure illustrates how trauma exposure gives rise to and perpetuates maladaptive interpretive frameworks, characterised by cognitive, emotional and behavioural patterns such as avoidance, dissociation, self-blame, and the formation of a trauma-related identity. These frameworks are further expressed through heightened emotional reactivity, social withdrawal, distrust, and hyperarousal, reinforcing the chronic adverse impact of trauma.

The array of negative interdependent and impairing symptoms and biopsychosocial disturbances ultimately foster a rigid pre-determined (e.g. *adverse meaning assigned in advance to stimuli*) and restricting over-determined (e.g. *adverse meaning assigned to stimuli is resistant to change*) outlook (Figure 1). The *pre-determined dimension* is characterised by the reflexive assignment of negative meaning to stimuli based on past traumatic experiences. This bias may lead individuals to anticipate danger or harm even in benign situations, filtering their perceptions through a lens of fear and mistrust. For instance, trauma survivors with PTSD may display an attentional bias towards threat, perceiving angry facial expressions as especially salient and exhibiting exaggerated physiological responses during fear learning and extinction, which reflects an ingrained expectation of repeated harm.^49^ These distortions are compounded by overgeneralisation, where innocuous stimuli are erroneously linked to past trauma, and by difficulties in recognising or integrating safety signals.^50^ Amygdala hyperactivity, impaired hippocampal contextual processing and diminished prefrontal regulatory control^51^ may represent the neurobiological underpinnings of this pre-determined framework and may further reinforce its persistence. Furthermore, these processes perpetuate affective dysregulation, avoidance and interpersonal withdrawal, thereby limiting sufficient engagement in intrapersonal and interpersonal dynamics that could foster corrective experiences. The persistent and restricting expectation of harm, combined with the avoidance of potentially restorative situations such as openness to new ideas, willingness to process affective states, forming trusting relationships, or engaging in adaptive behaviours, deprives individuals of crucial opportunities to challenge and reframe maladaptive cognitions and beliefs.

In contrast, the *over-determined* dimension highlights the rigidity and resistance of adverse interpretive processes to change, even when faced with contradictory evidence. These processes are characterised by repetitive and self-reinforcing cognitive patterns, such as trauma-related rumination, which involves persistent, negative thinking about the causes, consequences, and implications of traumatic experiences. Trauma-related rumination amplifies negative beliefs about the self (e.g. ‘I am broken’), others (e.g. ‘People are unpredictable and dangerous’), and the world (e.g. ‘Everything poses a potential threat’), entrenching these schemas in ways that make them resistant to modification. For instance, survivors may habitually replay past events in their minds, focusing on hypothetical scenarios (e.g. ‘What if I had done something differently?’) or engaging in self-blame (e.g. ‘All this is because of me’), which strengthens perceptions of helplessness and inadequacy. This perseverative thought process not only reinforces these beliefs but also increases emotional reactivity, as ruminative cycles heighten feelings of shame, guilt and fear. Over time, the over-determined dimension constrains cognitive and emotional flexibility, making it exceedingly difficult for individuals to develop novel, adaptive ways of thinking about their experiences. Instead, these processes further entrench the adverse beliefs that sustain the maladaptive interpretive framework, perpetuating cycles of dysfunction and impeding recovery.

The interplay of pre-determination and over-determination creates a self-reinforcing cycle characterised by a narrowing of experiential and psychosocial opportunities for positive change and growth. Pre-determined interpretations bias perceptions, priming individuals to detect and overreact to threats, while over-determined beliefs solidify these distortions, locking individuals into persistent symptoms such as hyperarousal, intrusive memories, and avoidance.

We argue that this conceptualisation is particularly relevant for emerging psychedelic therapies such as psilocybin treatment, which may hold promise in addressing the multifaceted nature of traumatic stress. Understanding how psilocybin may disrupt patients’ maladaptive interpretive framework can enable clinicians to harness its therapeutic potential and promote patient safety in future research studies.

## Psilocybin: Outcomes, mechanism of action and proposed psychological mechanisms of change across indications

Psilocybin, a naturally occurring psychedelic compound, has garnered significant interest for its potential therapeutic benefits. Studies have shown that psilocybin can facilitate profound psychological experiences characterised by heightened emotional processing and insight.^52,53^ These experiences, often described as mystical or transcendental, are believed to contribute to therapeutic outcomes by fostering a sense of interconnectedness and personal meaning.^54^ Psilocybin is a selective partial agonist of the 5-HT2A receptors, occasioning acute behavioural and psychological effects^55^ and promoting default mode network (DMN) modulation, a network of brain structures involved in self-referential processing and rumination.^56^ Classical psychedelics such as psilocybin are also thought to enhance synaptic plasticity and neurogenesis, which may occur due to the drug’s efficacy in activating the Gq second messenger pathway attached to the 5HT-2A receptor.^57^ Furthermore, the pharmacological disruption of the DMN is thought to promote psychological flexibility.^58^ In turn this may enhance learning processes by allowing patients to approach well-established, potentially maladaptive beliefs and associated behaviours from multiple perspectives, promoting divergent thinking, and insight. Recent evidence also highlights the potential role of brain-derived neurotrophic factor (BDNF) in psilocybin’s therapeutic effects.^59^ BDNF appears to be associated with key processes of adaptation, such as neurogenesis, synaptic plasticity, and long-term potentiation. Thus, psilocybin may facilitate remodelling of maladaptive cognitive and emotional patterns common in PTSD, particularly within a therapeutic context.^60^ This hypothesis is lent credence by the known association between trauma-related stress, hippocampal atrophy, and impaired neuroplasticity.^61^ By fostering neuroplasticity through these mechanisms, psilocybin may help modulate rigid trauma-related schemas and promote psychological flexibility.

Potentially mitigating experiential avoidance,^62^ psilocybin also facilitates emotional breakthroughs, allowing patients to process repressed or previously avoided emotions.^52^ Furthermore, experiences of ego-dissolution have been widely reported, often promoting a sense of unity and interconnectedness, reducing feelings of isolation and fostering a greater sense of belonging.^63^ Correspondingly, a subset of psychedelic trial participants report indirect forms of trauma processing, where traumatic memories and emotions were addressed indirectly during their treatment, at times accompanied by beneficial, immersive and transcendental states.^22^ Lastly, some participants describe processes characterised by reorganisation of self-narratives following psychedelic treatment, reporting changes in their self-perception through processes of relatedness and identification with the psychedelic experience, themselves, and significant others.^22^ Taken together, these findings suggest that psilocybin could be a promising treatment for PTSD, warranting further rigorous research to establish its safety and efficacy and explore its mechanisms of action.

## Disrupting PTSD in psilocybin treatment

Given the novelty of psilocybin as a treatment for PTSD and the many unknowns, this section examines how psilocybin may promote positive outcomes (Table 1). The discussion aims to inform future research on its safety, efficacy, underlying mechanisms and patient experiences.

**Table 1.** Disrupting PTSD in psilocybin treatment.

Targeting dysregulated biological fear responses	Potential mechanism	Description
	Modulation of threat perception and amygdala reactivity^51^	• Psilocybin appears to reduce excessive attribution of significance to perceived threats by modulating salience network connectivity. • Psilocybin also influences cortical-midline structures, potentially improving fronto-parietal regulation of the amygdala and reducing hyperreactivity to negative stimuli. • Psilocybin decreases amygdala activity, improving emotional regulation and thereby potentially mitigating exaggerated fear responses and hyperarousal in PTSD.
	Enhancement of prefrontal-amygdala connectivity^51^	• Psilocybin may enhance bidirectional connectivity between the amygdala and medial prefrontal cortex (mPFC), a mechanism believed to be critical for regulating emotional responses. Improved connectivity in this network could facilitate more effective top-down modulation of fear responses and contribute to the restoration of emotional regulation, which is often disrupted in PTSD.
		• By potentially strengthening functional connections between the mPFC and limbic structures, such as the amygdala and hippocampus, psilocybin may help reduce hypervigilance, intrusive memories, and emotional dysregulation, thereby supporting more adaptive emotional processing and resilience.
	Facilitation of fear extinction and enhancement of neuroplasticity^51^	• Psilocybin may promote the extinction of conditioned fear responses, a process considered essential for trauma recovery. Preclinical studies indicate that psilocybin could enhance fear extinction by influencing molecular pathways associated with neuroplasticity and modulating activity within fear-related neural networks.
		• Psilocybin appears to reduce amygdala reactivity, potentially diminishing the emotional intensity of trauma recall. Neuroimaging findings suggest that this attenuation of amygdala hyperactivity may improve emotional regulation, creating opportunities for trauma reprocessing with reduced distress. • Psilocybin has been shown to increase neuroplasticity markers, including dendritic growth and synaptic density, particularly in the prefrontal cortex. These changes may contribute to the remodelling of neural circuits involved in emotional memory processing and adaptive regulation of trauma-related responses.
Modulating excessive use of psychological defences	Potential mechanism	Description
	Mitigating avoidance responses and behaviours^62,64^	• Psilocybin may promote a therapeutic state that enables patients to confront and process trauma-related material previously avoided.
		• By reducing experiential avoidance of trauma-related affect, somatic states, cognitions, and memories, psilocybin may support patients engage with highly distressing experiences in a safe and controlled setting.
	Addressing dissociative symptoms^51,64^	• By recalibrating activity in the vmPFC and dACC, psilocybin may help reduce dissociative symptoms such as depersonalisation and emotional numbing.
	Counteracting denial and minimisation	• Psilocybin may attenuate denial by enhancing self-referential processing in brain networks like the default mode network (DMN).^65^ • Promoting introspection,^66^ this may facilitate access to suppressed trauma-related material for patients, fostering acknowledgement of their lived experience and supporting more adaptive psychological processing.
Challenging negative cognitive appraisals and harmful self-narratives	Potential mechanism	Description
	Reducing negative self-referent cognitions	• Cognitive flexibility and recalibration: Psilocybin could disrupt rigid cognitive patterns in the Default Mode Network (DMN), reducing maladaptive beliefs and creating an optimal window for cognitive restructuring.^67^ • By enhancing psychological flexibility,^68^ psilocybin may help patients break cycles of rumination, alleviating shame and self-blame while fostering healthier, more adaptive self-perceptions.
	Expanding sense of self and beliefs about self, others and the world^69^	• Psilocybin may facilitate an expanded sense of self, allowing patients to re-evaluate and reconceptualise their identity beyond the constraints of trauma. • This broader perspective may promote the formation of more adaptive beliefs about self, others, and the world.
	Disrupting rigid and harmful trauma-related self-narratives and implicit trauma identity	• Psilocybin appears to facilitate shifts in perspective and deepen insight, potentially allowing patients to re-evaluate rigid trauma-related narratives that reinforce an implicit trauma identity.
		• By helping individuals reconsider the relationship between their sense of self and their traumatic experiences, psilocybin could reduce the burden of maladaptive self-perception.
Remediating dysfunctional interpersonal and psychosocial dynamics	Potential mechanism	Description
	Enhancing connectedness and relational functioning^70^	• Psilocybin may help patients cultivate feelings of connectedness to self, others, and the broader world, promoting empathy and a sense of belonging. • These effects may help PTSD patients rebuild interpersonal trust and develop healthier relational behaviours and attitudes.
	Disrupting painful relational patterns and rebuilding trust	• By increasing self-awareness and self-compassion,^71^ psilocybin may help patients recognise repetitive patterns of relational dynamics.
		• Additionally, the treatment process may provide a positive experience of care in a vulnerable state, which may help participants rebuild interpersonal trust and safety.
	Reducing isolation and social withdrawal^72^	• Psilocybin’s potential prosocial effects may mitigate social withdrawal and loneliness by fostering a sense of community and belonging, whether through interpersonal relationships, broader social interactions, spiritual practices, or connection with nature. • These shifts may support PTSD patients to reintegrate into social networks and promote greater psychosocial well-being.

dACC, dorsal anterior cingulate cortex; PTSD, Post-traumatic stress disorder; vmPFC, ventromedial prefrontal cortex.

### Targeting dysregulated biological fear responses

PTSD is marked by dysregulated fear responses and heightened sensitivity to perceived threats, resulting in exaggerated startle responses, hyperarousal and heightened emotional reactivity.^44^ These are linked to an overactive amygdala, the brain region responsible for processing fear and emotional responses, as well as a dysregulated hypothalamic-pituitary-adrenal (HPA) axis, which governs the body’s stress response.^73^ PTSD is characterised by distinct HPA axis dysregulation, including hypocortisolism, where reduced baseline cortisol levels occur despite elevated corticotropin-releasing hormone (CRH) concentrations.^74^ This paradox reflects heightened glucocorticoid receptor (GR) sensitivity and enhanced negative feedback, leading to suppressed cortisol production. Such hypoactivity compromises cortisol’s anti-inflammatory role, contributing to chronic low-grade inflammation and systemic homeostatic disruption. This dysregulation is further implicated in PTSD-related physical comorbidities, such as cardiovascular disease, autoimmune conditions, and metabolic disorders.^75^ Moreover, emerging evidence suggests that the periaqueductal grey (PAG) plays a critical role in mediating defensive reactions, particularly in dissociative subtypes of PTSD.^76,77^ While hyperarousal presentations are often driven by amygdala overactivity and a hypervigilant fight-or-flight response, the PAG is central to the ‘freeze’ response (e.g. an automatic survival mechanism activated in situations of extreme or inescapable threat). This distinction underscores the heterogeneity of PTSD and highlights the neurobiological complexity of dissociative symptoms, such as emotional numbing, depersonalisation, and derealisation.

Correspondingly, psilocybin appears to modulate these neurobiological and cognitive processes underlying dysregulated responses. By altering salience network connectivity, psilocybin may influence threat perception and potentially reduce the overattribution of significance to perceived dangers.^51^ Additionally, psilocybin may help balance activity in cortical-midline structures, potentially strengthening the fronto-parietal control over amygdala reactivity^51^ and decreasing amygdala reactivity to negative emotional stimuli while enhancing connectivity between the amygdala and prefrontal cortex, a mechanism critical for regulating emotional responses.^51^ These effects may facilitate fear extinction learning, allowing patients to reprocess traumatic memories and reduce their emotional intensity.

### Modulating excessive use of psychological defences

Individuals with PTSD often rely on excessive psychological defences to cope with distressing memories and emotions.^78,79^ These defences may include avoidance, projection, denial and minimisation and dissociation. Avoidance behaviours, such as steering clear of situational reminders of the traumatic event or phobic avoidance of traumatic memories, can prevent individuals from confronting and processing their index trauma, thereby maintaining PTSD symptoms.^80^ Furthermore, structural dissociation, a core feature of PTSD and trauma-related conditions,^81^ arises from the brain’s inability to fully process and integrate overwhelming traumatic experiences. Instead of forming cohesive, trauma-related narratives, different aspects of the traumatic event, including affective states, physical sensations and trauma-related cognitions may become fragmented and segregated within distinct memory networks. This fragmentation contributes to a disjointed sense of self, disruptions in memory, and a range of dissociative symptoms, including affective numbing, traumatic amnesia and a sense of detachment or depersonalisation.^82^ Importantly, dissociation could also be conceptualised as a defence mechanism, allowing individuals to cope with overwhelming experiences by psychologically distancing themselves from traumatic events.^83^ However, persistent dissociation can significantly impair daily functioning, relationships, and overall well-being. For instance, individuals exposed to trauma often struggle to accurately assess their safety in various contexts, leading to chronic feelings of threat and hypervigilance. This phenomenon, termed “contextualisation deficit” is associated with overactivity in the dorsal anterior cingulate cortex and underactivity in the ventromedial prefrontal cortex, which are brain regions involved in regulating fear and craving responses.^84–86^

Psilocybin appears to enhance connectivity within brain networks associated with emotional and self-referential processing,^87^ potentially fostering a broader sense of self and interconnectedness. This enhanced connectivity may enable patients to reframe their traumatic experiences and gain a deeper understanding of the contexts in which those events occurred. Similarly, self-blame, helplessness and feelings of worthlessness further exacerbate the condition by reinforcing negative self-perceptions.^88,89^ Psilocybin has been shown to facilitate emotional breakthroughs and may reduce the use of maladaptive defences including experiential avoidance.^52,62^ The immersive altered state induced by psilocybin, characterised by heightened and novel sensory perception, enhanced introspection, profound emotional experiences, and a dissolution of ego boundaries may support patients to directly and indirectly confront and process a range of avoided memories and effects in a safe, trustworthy and controlled therapeutic setting. In turn, this process can potentially lead to a reduction in avoidance behaviours and dissociative symptoms, allowing for more effective engagement with trauma material.

### Challenging negative cognitive appraisals and harmful self-narratives

Subjective narratives of traumatic experiences can become central to patients’ identities, negatively shaping their perceptions of themselves, others and the world.^90,91^ Trauma-related narratives are characterised by persistent negative affect and cognitions, including catastrophic thinking, worthlessness and hopelessness. Over time, these narratives can form and solidify an implicit trauma identity,^92–94^ making it difficult for patients to separate their sense of self from their traumatic past. Therefore, trauma narratives and self-narratives may converge into a singular, intertwined negative self-image and identity. Interpersonal TE, including childhood sexual abuse and domestic violence can also involve betrayal, manipulation and secrecy.^95,96^ Patients report high shame and interpersonal distrust that may further consolidate negative self-referent cognitions, adversely impacting patients’ capacity to form and maintain relationships.^97,98^ Leading to significant psychosocial deficiencies, traumatised patients report isolation and withdrawal.^99,100^

Psilocybin and other psychedelics could offer several potentially beneficial mechanisms that may benefit PTSD patients, including enhanced neuroplasticity.^101^ More specifically, psilocybin treatment may help disrupt harmful, trauma-related self-narratives by promoting cognitive flexibility and insight. For example, the mystical and transcendental experiences reportedly induced by psilocybin^102^ can lead to a re-evaluation of self-identity and a renewed sense of purpose and meaning. Potentially facilitating an expanded sense self and beliefs about self, others and the world, these experiences may be able to help patients develop more adaptive and positive self-narratives. Similarly, psychedelics may also disrupt rigid cognitive patterns and negative self-appraisals, including guilt and shame, that maintain PTSD.^71,103,104^ By promoting psychological flexibility and self-compassion psilocybin may be able to help patients reframe their experiences, reduce rumination, and develop more adaptive beliefs about themselves and the world. These cognitive shifts can be pivotal in breaking the cycle of negative thoughts and behaviours that sustain PTSD.

### Reducing dysfunctional psychosocial dynamics

PTSD often results in dysfunctional interpersonal and psychosocial dynamics, including repeating painful relational patterns, shame, distrust, irritability, and social withdrawal. These dynamics are marked by difficulties in forming and maintaining healthy relationships and a pervasive sense of isolation.^105^ These may be, in part, attributed to relational traumatic re-enactments that involve unconsciously recreating aspects of the trauma in current relationships, leading to repeated cycles of distress and conflict.^106^ Distrust and irritability further complicate interpersonal interactions, while social withdrawal exacerbates feelings of loneliness and alienation.^107^ Psilocybin and other psychedelics have been found to enhance feelings of connectedness to self, others and the natural world, which can improve interpersonal and relational functioning.^108–110^ By fostering prosocial interpersonal feelings and dynamics, including empathy, compassion and a sense of belonging to a broader community, spiritual traditions or the land, psilocybin may help PTSD patients rebuild interpersonal trust and develop healthier relational behaviours while reducing isolation and social withdrawal. Furthermore, qualitative reports from psychedelic trials across indications suggest that a subset of participants experience significant intrapersonal corrective shifts during and after treatment sessions.^22^ These participants describe transformative moments in which they can process unresolved interpersonal conflicts and access new relational insights, leading to an increased ability to view their relationships in healthier and more constructive ways. For many individuals adversely affected by interpersonal forms of TE, these processes may facilitate a sense of interpersonal resolution and understanding that positively influences their current relationships and overall psychosocial functioning.

### Safety and future research recommendations

As research advances in PTSD neurobiology, there is also increasing recognition of how adverse psychosocial factors contribute to trauma-related conditions, including PTSD, C-PTSD and dissociative disorders (REF). While preliminary findings suggest psilocybin may hold therapeutic potential in TE populations, ethical and safety considerations remain paramount, particularly given the lack of research specifically addressing PTSD patients. Psilocybin treatment outcomes are also influenced by non-pharmacological factors, such as treatment setting and rapport, which have long been central to psychedelic therapy.^111,112^ Established principles in psychedelic research emphasise set and setting alongside structured preparatory and integration sessions to improve safety and efficacy.^113^ However, these protocols may require adaptation to trauma-specific needs, as individuals with trauma histories could face increased risks of affective dysregulation, dissociation, and re-traumatisation in immersive psychedelic experiences.

Given the largely unexplored role of psilocybin in PTSD treatment, careful attention should be given to factors that might shape treatment response, including the nature of traumatic experiences (e.g. interpersonal vs non-interpersonal trauma) and pre-existing intrapersonal and psychosocial vulnerabilities impacting functioning. While comparative research is still lacking, trauma-informed adaptations within existing protocols may help minimise risk and enhance therapeutic outcomes. To advance ethical research and clinical application, this section identifies key safety parameters and foundational recommendations, underscoring the necessity of rigorous, trauma-sensitive protocols that safeguard participant well-being while optimising the potential benefits of psilocybin treatment for trauma survivors.

### Screening processes for patient trauma history

Implementing comprehensive, trauma-sensitive screening protocols may enhance safety in psychedelic therapy research. Childhood maltreatment, interpersonal violence and non-interpersonal trauma are closely linked to difficult-to-treat conditions such as personality, mood, anxiety, and substance use disorders.^114,115^ The impact of TE varies: childhood maltreatment is associated with a higher risk of mood and anxiety disorders, while lifetime interpersonal trauma appears to increase susceptibility to substance use disorders.^116^ Given this variability, psychedelic screening approaches should consider how different trauma types influence personality organisation, affect regulation, and attachment patterns to improve patients’ experience. Investigators are increasingly recognising individual differences in psychedelic treatment outcomes.^113,117^ Therefore, integrating trauma-sensitive screening methods may help identify patients with unresolved distress, assessing not only medical eligibility but also psychological suitability and readiness. Below, we outline key considerations for enhancing patient safety.

#### Single event adult trauma versus cumulative childhood trauma

Cumulative childhood trauma (CCT) is often associated with more severe and persistent symptomatology, including affect dysregulation, chronic shame, identity fragmentation and interpersonal difficulties.^118,119^ Traditional PTSD treatments, particularly exposure-based approaches, may not always address these complexities, potentially leading to an inadequate treatment response and increasing the risk of retraumatisation.^97^ Additionally, many diagnostic frameworks do not fully account for neurobiological dysregulation or the developmental consequences of early trauma, highlighting the need for phase-based stabilisation before engaging in trauma processing. In contrast, single-event adult trauma (SEAT), while deeply impactful, tends to present with less complex psychopathology. Although survivors may experience hyperarousal, intrusive memories, and avoidance, their self-structure is often more intact.^97^ Distinguishing between CCT and SEAT can enhance safety by tailoring interventions to the unique needs of each population. CCT survivors may benefit from a gradual, stabilisation-focused approach during preparation, aimed at addressing disturbances in self-organisation and difficulties with establishing rapport. In contrast, SEAT survivors are often better positioned to engage in trauma processing more directly and with fewer preparatory requirements.

#### Timing and readiness for trauma-focused interventions

The timing of intervention plays a key role in PTSD treatment.^120^ Clinical guidelines emphasise the importance of assessing symptom persistence to distinguish transient stress responses from chronic psychopathology. The UK’s NICE guidelines recommend a period of watchful waiting for mild cases within the first 4 weeks, followed by reassessment to monitor natural recovery,^121^ while in the US, early intervention is encouraged for acute stress disorder to reduce the likelihood of PTSD progression.^122^ Factors influencing treatment readiness include symptom severity, the likelihood of natural recovery, psychological preparedness and social support. A phase-based stabilisation approach may be particularly relevant for individuals experiencing ongoing trauma exposure (e.g. domestic violence, active military deployment) or acute safety concerns, such as suicidality or self-harm. For psilocybin treatment, evaluating trauma recency, frequency and dissociative symptoms could be important in determining suitability. Patients with recent trauma (within 6–12 months) may not yet be in a sufficiently stable psychological state to engage with the potentially intense experiential nature of the treatment. Screening should also consider ongoing legal conduct and a thorough evaluation of the patient’s current psychosocial support system, including whether they have disclosed their traumatic experiences to significant others and whether there is an ongoing risk of TE.

#### Promoting safety through trauma-informed preparation

Trauma-focused preparation practices may help improve safety and readiness for psilocybin treatment. In addition to standard psychedelic trial protocols (e.g. rapport-building, psychoeducation, grounding techniques, intention-setting), trauma-specific preparation could include:

- **Psychoeducation**: Providing information on the neurobiological effects of trauma, including activation of the fight-flight-freeze response and its impact on the amygdala, hippocampus and prefrontal cortex, may help participants normalise their symptoms and reduce self-stigma. Given that relational distrust and anxiety about accessing care are common among trauma survivors,^98,123^ explicit communication from the treatment team regarding safety, boundaries, and treatment procedures may foster a greater sense of security. Psychoeducation should also emphasise that psilocybin treatment differs from other treatments, as it encompasses a wide range of subjective experiences. While some participants may engage directly with trauma-related content, others may primarily encounter affective, somatic and/or non-dual states without specific memory recall. Setting clear expectations about this variability may reduce fear and enhance trust in the process. To promote safety, investigators should encourage patients to remain present with their subjective experiences without actively guiding them towards traumatic memory retrieval unless such material emerges organically. Given the early stage of research into PTSD and psychedelic therapy, a non-directive, patient-centred approach may help prevent distress and minimise the risk of retraumatisation. Avoiding overtly exposure-based methods will allow for continued exploration of how PTSD patients respond to psilocybin treatment, balancing engagement with trauma-related material while ensuring that the process remains non-coercive and empowering.
- **Boundary setting and safety planning:** For TE individuals, disruptions in boundary formation can contribute to heightened vigilance, distrust or dependency.^124^ During treatment, attachment-related responses may present as excessive reassurance-seeking, difficulties with autonomy, or withdrawal in response to perceived relational threats.^125^ To mitigate these risks, investigators should establish clear role definitions, ethical boundaries, and scope of support, ensuring that participants understand the distinction between clinical trials and standard psychiatric care. Investigators should encourage participants to articulate specific trauma-related needs or concerns, treatment expectations, and understand the study limitations, particularly in relation to long-term mental health care. Furthermore, clarifying the differentiation between clinical research protocols and community-based psychiatric services may prevent misinterpretation of therapeutic boundaries and reduce the likelihood of reactivating attachment-related distress. Implementing structured boundary-setting measures can support treatment adherence, minimise distress, and facilitate a more stable therapeutic engagement throughout the intervention. Lastly, the treatment environment should be designed to reduce risks of retraumatisation. A calming and secure physical setting is essential, allowing patients to explore their reactions in a supportive environment. Particular attention should be paid to ensuring the setting is free of objects, smells or other sensory stimuli that could inadvertently trigger associations with the participant’s index trauma. Additionally, the therapists’ positioning during sessions should be thoughtfully considered, with participants encouraged to express preferences regarding therapist proximity to optimise their sense of safety and comfort.
- **Personality and defence mechanisms**: Individual traits and psychological defences may influence responses to psychedelic experiences.^126^ For instance, individuals with high neuroticism could be more prone to negative responses,^127^ suggesting a need for targeted preparation. Conversely, those high in openness may benefit from expectation management to ensure their therapeutic goals are well-aligned. Psychological defences should be understood and discussed with TE patients as adaptive mechanisms rather than pathological traits, serving a role in maintaining psychological stability following TE. Avoidance, control, dissociation and hypervigilance often function as self-protective strategies and may become particularly salient during psilocybin treatment.^128^ Rather than being conceptualised as barriers to safety and meaningful treatment engagement, these responses should be contextualised within the individual’s trauma history and normalised as part of their coping repertoire. Encouraging patients to acknowledge and explore these mechanisms within a structured, safe clinical environment may promote deeper engagement with the treatment process.

#### Sex, gender, race and culturally based differences in trauma

It may be equally important to consider how sex, gender, race, and cultural background influence responses to traumatic experiences and treatment choice and response.^129–131^ These variables can also influence perceptions of and attitudes towards subjective experience of psychedelics.^131^ In turn, psilocybin treatment should be delivered with an awareness of these factors as differences in neurobiological, psychological, and sociocultural responses to trauma may alter how individuals experience both the therapeutic process and its outcomes. Currently, there is no empirical data to inform specific, tailored approaches to psilocybin treatment that account for these factors, and it is beyond the scope of this review to provide detailed recommendations. Nevertheless, with the aim of enhancing safety and inclusivity in psilocybin treatment protocols, we highlight these differences and offer brief and generalised examples of how such variables might be addressed in clinical research.

#### Sex and gender

While trauma can affect individuals across all demographics, sex and gender significantly influence trauma exposure, symptomatology, and treatment responses.^129^ Sex refers to biological differences between males and females, whereas gender encompasses social and cultural roles, behaviours and identities.

Epidemiological studies indicate that men are more likely to experience trauma overall, however, women have higher rates of interpersonal trauma (e.g. physical abuse, sexual assault, domestic violence), which carries the highest conditional risk for PTSD.^132^ Specifically, women survivors of sexual assault often experience guilt, shame, anger, and self-recrimination, which can affect treatment engagement.^133^ Societal factors, such as rape myths and victim-blaming, further exacerbate distress, contributing to isolation and demoralisation.^134^ Additionally, women are more likely to experience early-life trauma, which can disrupt developmental processes and increase their vulnerability to chronic trauma-related disturbances.^135^ Gender also influences PTSD symptomatology and coping mechanisms. Women may exhibit higher rates of depression, dissociation, and somatic complaints, while men more frequently present with anger, aggression, and externalising behaviours, including substance use disorders.^136^ Socialisation patterns shape how trauma is processed and expressed, with men often discouraged from emotional vulnerability and encouraged towards stoicism and self-reliance.^137^ These norms may delay help-seeking and contribute to early dropout from PTSD treatments, aligning with findings of higher attrition rates among men.^138^

Therefore, gender-responsive, trauma-informed approaches may improve engagement and safety in psilocybin treatment. Psychoeducation should acknowledge gender differences in trauma responses, helping to counter stigma, normalise experiences, and reduce shame. This may foster openness to altered states induced by psilocybin, potentially increasing treatment receptivity. Additionally, therapist–participant pairings should be considered to minimise relational dynamics that may contribute to destabilisation or distress. Women survivors of sexual violence, for example, may hesitate to express preferences for a therapist’s gender, particularly when assigned male therapists. Actively involving participants in therapist selection and emphasising psychological safety, clear boundaries and collaborative decision-making may enhance trust and treatment engagement, particularly for those with histories of prolonged interpersonal trauma.

#### LGBTQIA+ and BIPOC populations

LGBTQIA+ individuals face high rates of TE, mental health challenges, and systemic discrimination, which may foster distrust towards providers.^139,140^ Racial trauma profoundly impacts the mental, emotional and physical health of Black, Indigenous and People of Colour (BIPOC) through systemic and interpersonal racism. Black individuals experience disproportionate levels of anti-Black racism (ABR), leading to psychological injuries with symptoms resembling post-traumatic stress disorder (PTSD), such as nightmares, hypervigilance and avoidance.^141^ Furthermore, experimental trials and psychedelic studies indicate substantial underrepresentation of minorities and BIPOC compared to non-Latino White populations,^142,143^ emphasising the need for greater inclusion. To address these barriers, culturally attuned recruitment, assessment and retention strategies should be implemented in psychedelic PTSD studies.^144^ Diversifying research teams to reflect participants’ cultural and identity backgrounds may foster trust and improve communication.^145^ Research protocols should also prioritise inclusivity by adapting flyers and recruitment materials with culturally relevant language and visuals, taking into consideration attitudes towards psychedelics and trauma to ensure they resonate with local, diverse communities. Treatment settings should be affirming and inclusive, incorporating gender-neutral facilities and culturally representative artwork, music and therapeutic approaches.^145^

#### Military veterans

Military veterans with PTSD face distinct biopsychosocial challenges influenced by combat trauma, military culture, and pre-enlistment adversity.^146,147^ Despite increased efforts to improve care, veterans engage in mental health treatment at lower rates than civilians, citing stigma, mistrust, physical disabilities, and cultural barriers.^148,149^ Some may also feel alienated from civilian clinicians unfamiliar with military culture, reinforcing reluctance to engage in therapy.^150^ Additionally, the transition to civilian life presents challenges, including identity loss, loneliness and moral injury, where perceived violations of personal ethics exacerbate guilt and shame.^151,152^ Many veterans with PTSD also experience chronic pain, psychiatric comorbidities and psychosocial dysfunction, further complicating treatment.^153,154^

To improve outcomes, clinicians should receive training in military-specific trauma and moral injury to build rapport and improve engagement.^155^ Including veteran peer mentors in trials may foster trust and reduce stigma, encouraging participation. Expanding telehealth options for screening, preparation and integration can also enhance accessibility, particularly for rural or remote veterans. Furthermore, group therapy formats and peer support may provide a scalable approach to psilocybin treatment while reinforcing camaraderie and peer support known to support treatment engagement among veterans.^156^

Taken together, the heterogeneity of TE and its adverse impact on individuals’ biopsychological makeup may significantly influence patient responses to psychedelics. TE individuals may be more vulnerable or sensitive to the adverse effects associated with psychedelics, including transient anxiety, confusion, or physical discomfort, potentially leading to exacerbation of symptoms. Therefore, incorporating detailed trauma histories into routine eligibility assessments in psychedelic studies can help identify individuals at risk of disorder transitions and intervention suitability, thereby enabling the customisation of psychological support offered to trial participants.

### Trauma-sensitive informed consent

The emphasis on enhancing informed consent processes is a response to the risk of retraumatisation and the possibility of destabilising and unpredictable perceptual and psychological effects and changes during and after psychedelic treatment. TE may increase the potential for retraumatisation when accessing care by disrupting patients’ ability to trust and accurately assess danger, leading to relational distrust, impaired decision-making and increased vulnerability to subsequent victimisation.^157^ The cyclical nature of TE and retraumatisation, where each new traumatic event may exacerbate the effects of previous ones, underscores the need for interventions that prioritise the restoration of a clinically meaningful sense of safety and the promotion of trust in self, others and world. In turn, psychedelics can induce acute perceptual changes, ego dissolution, and altered metaphysical beliefs that may persist beyond the immediate experience.^158,159^ Such experiences, while therapeutic for some, can also be disturbing and disorienting, potentially leading to adverse outcomes, including increased suicidal ideation, elevated anxiety or persistent perceptual disturbances.^160^ Together, there may be a heightened risk of TE patients to experience intensified feelings of vulnerability, suggestibility and anxiety before, during and after treatment. The lack of research investigating the impact of TE on the nature, trajectory and outcome of psychedelic treatment underscores the importance of enhanced informed consent in psychedelic research.

Enhanced informed consent processes that address the potential adverse sequelae of TE could help ensure that participants are fully aware of the potential challenges, benefits, and the unpredictable nature of psychedelic treatment. To enhance informed consent processes, researchers should consider adopting a variety of approaches and best practices.^161–164^ These may include thorough preparation that fosters trust, dynamic consent, simplified language, digital health tools, the breakdown of complex information into smaller sections, and the use of visual aids or interactive questioning to ensure comprehension. Participants should also be informed about the potential for prolonged effects and the variability in individual responses. Importantly, TE is associated with impairments in attention, memory, executive functions, and other cognitive domains.^165^ Therefore, continuous consent should be obtained throughout psychedelic trials of trauma-related conditions, especially since participants’ cognitive capacities may modulate due to the intense subjective effects catalysed by psychedelics. Furthermore, as patients’ will have likely attempted a range of standard treatments, including medications and exposure-based, cognitive behavioural therapies, it is important that prospective participants fully understand and agree to the novel, psychedelic treatment process. Crucially, highlighting the rationale for the deployed therapeutic approach and how it differs from evidence-based PTSD treatments in terms of processing of traumatic material may support patients develop a sense of agency and autonomy, rebuild trust in clinical teams and self, reduce anticipatory anxiety, and foster a collaborative interpersonal dynamic to promote safety outcomes. By enhancing informed consent procedures, tailored to the unique presentation of traumatised individuals and the specific elements of psychedelic treatment, researchers can uphold ethical standards and mitigate the potential for retraumatisation, potentially leading to psychiatric destabilisation and adverse events.

### Mitigating re-enactments and managing power dynamics

The potential for re-enacting painful and unhelpful relational dynamics during psychedelic treatment may be heightened, especially in PTSD patients. Re-enactments refer to processes of conscious and unconscious repetitions of past painful or traumatic experiences in an individual’s everyday life.^106^ Trauma survivors may involuntarily recreate situations that mirror their original trauma, such as engaging in relational dynamics or behaviours that repeat the adverse characteristics of past, traumatic experiences. These re-enactments in routine practice, if not properly managed, can lead to further psychiatric destabilisation.^166,167^ For instance, trauma survivors may be at risk for retraumatisation, particularly in therapeutic settings where power dynamics can mirror past abuses. In healthcare, this can manifest when clinicians unknowingly replicate dynamics of abuse, such as minimising autonomy or facilitating a maladaptive state of dependence. The potential for adverse power dynamics is particularly enhanced in relationships where the clinician holds significant authority, which can inadvertently reinforce the patients’ sense of helplessness. This may be highly pertinent to psychedelic treatment, where trial participants are significantly dependent on the clinical team for safety due to the disorienting and powerful effects of psychedelics.

To mitigate the potential for harmful re-enactments and adverse relational power dynamics, research teams should consider adopting practices that prioritise patient autonomy and empower survivors.^123^ This includes ongoing sensitivity to power imbalances, a collaborative approach to goal setting, and ensuring that participants have a subjective sense of control over the pace and direction of the intervention. Acknowledging power differentials and the potential for intense treatment processes, psychedelic research protocols should address the limited and safe use of supportive physical touch, elevated patient vulnerability, and the importance of provider monitoring and supervision. By addressing these factors, researchers can create safer environments that reduce the risk of retraumatisation.

### Standardising care by promoting consistency

Manualised approaches to standard PTSD treatments promote consistency and adherence to evidence-based psychological interventions, enhancing treatment safety, effectiveness and replicability.^168^ These structured treatment protocols guide clinicians through established procedures and techniques, reducing variability and improving patient outcomes in face of often challenging therapeutic processes. Similarly, robust ethical and clinical guidelines in psychological treatments are vital, safeguarding patient rights, promoting transparency and providing a framework for monitoring and supervision of clinicians delivering care. Given the complexity and heterogeneity of PTSD symptomatology, coupled with the largely unpredictable and potentially challenging nature of psychedelic treatment, encompassing participants’ subjective responses to the elicited psychedelic effects, manualised psychedelic treatment protocols and ethical guidelines are essential for patient safety. The uniformity of treatment protocols and ethical guidelines offers a coherent clinical and theoretical framework, improving care delivery, enabling meaningful clinical supervision and allowing for a systematic approach to data analysis, crucial for regulatory approval processes and monitoring.

However, across TE and non-TE patient populations, manualised psychological treatments are not necessarily superior to non-manualised interventions.^169–171^ For example, the non- and self-directed methods employed in various forms of psychedelic therapy appear to provide unique therapeutic opportunities that are not typically available through standard, highly manualised trauma-focused treatments.^22^ Some patients may benefit from more exploratory, psychodynamic, interpersonal, psychospiritual or psychosocial interventions as an adjunct to psilocybin treatment. Nevertheless, given the novelty of psilocybin research in PTSD, future trials should aim to standardise therapeutic approaches that are first and foremost responsive to psilocybin’s acute effects while fostering participants’ sense of safety and agency to enhance their capacity for inner exploration and immersion in the psychedelic experience.

### Optimising clinical training

Specialist training of therapists supporting participants in psychedelic studies is standard.^172,173^ In the context of traumatic stress, as psychedelic treatment may evoke intense emotional responses and enable access to previously avoided material, without adequate training, providers may fail to provide the necessary support during treatment, unintentionally exposing patients to re-traumatisation and re-victimisation. To address these challenges, supplementing current training protocols with trauma-informed practices could support research teams and participants achieve better safety outcomes. First, therapists would benefit from a foundational understanding of the wide-ranging adverse biopsychosocial effects of TE, as well as how psychedelics may interact with these processes. For example, providers should be capable of providing support that acknowledges the non-pathological function of trauma responses, including experiential avoidance, excessive need for control, anxiety reactions due to disturbing somatic states and interpersonal distrust. Given, the broad phenomenological nature of psychedelic states encompassing somatic, interpersonal, psychological and spiritual dimensions^174,175^ and the inherent diversity of trauma responses,^176^ viewing these as potentially clinically meaningful, treatment-catalysed processes requiring exploration rather than elimination may support participants engage with the more challenging psychosomatic states potentially involved in treatment. Second, training of therapists and clinical research organisations should emphasise the development of strong, positive interpersonal dynamics based on transparency, collaboration, trust, safety and patient empowerment. Given that PTSD patients often struggle with trust due to forms of betrayal associated with interpersonal forms of TE,^177^ therapists and research sites must be skilled in creating secure clinical environments by providing predictable and consistent experiences of care. This involves recognising and responding to signs of distress arising from adverse interpersonal dynamics as well as being adept at stress management techniques, which can help patients stay present and engaged during difficult treatment sessions. Moreover, providers should receive training in recognising and managing their own emotional responses to patients’ material. Regular mentoring or supervision, peer debriefing and self-care practices should be integral components of training programs to help therapists maintain their own mental health while providing effective care to traumatised patients in psychedelic trials.

## Conclusion

The conceptualisation of PTSD in Figure 1 highlights the complex network of factors that sustain the condition. Psilocybin may represent a potential novel therapeutic approach, facilitating the disruption of maladaptive interpretative frameworks related to self, others, and the world. By targeting dysregulated fear responses, modulating psychological defences, challenging harmful self-narratives, and improving interpersonal dynamics (Table 1), psilocybin treatment could enhance existing PTSD interventions. However, current evidence remains preliminary, largely drawn from preclinical research and early-phase trials, necessitating further investigation.

Future research should consider empirical validation of these hypotheses, focusing on the development of trauma-informed protocols that prioritise participant safety. Investigators may need to address the impact of TE, refine informed consent processes and adopt trauma-sensitive practices to reduce risk and improve outcomes. It will also be valuable to explore how trauma history and psychopathology influence responses to psychedelics, using psychometric tools and qualitative methodologies to assess the relationship between traumatic stress symptoms and psychedelic experiences. The neurobiological, psychosocial and phenomenological dimensions discussed in this manuscript could inform the design and delivery of structured psychological support during psilocybin treatment. This approach could enhance participant safety, improve provider training and establish consistent care practices across clinical research settings. Further refinement of trauma-informed procedures may improve feasibility and safety across populations with varying PTSD subtypes.

Once sufficient safety and efficacy data have been generated, future studies could explore integrating evidence-based interventions or traditional psychotherapies with psilocybin treatment. These interventions could be delivered in the weeks surrounding the psilocybin treatment session, leveraging existing protocols and trained personnel to improve access, enhance outcomes, and promote standardisation in clinical practice. Developing standardised protocols may also help identify predictors of treatment response and enable the creation of tailored, patient-centred treatment plans, ultimately contributing to more effective and personalised care for individuals with PTSD.

## Declarations

**Ethics approval and consent to participate:** Not applicable.

**Consent for publication:** Not applicable.

**Availability of data and materials:** Not applicable.
